# Food safety culture in food companies amid the Lebanese economic crisis and the Covid-19 pandemic

**DOI:** 10.1016/j.heliyon.2023.e19885

**Published:** 2023-09-09

**Authors:** Zeina Nakat, Vera Tayoun, Samar Merhi, Christelle Bou-Mitri, Layal Karam

**Affiliations:** a Faculty of Nursing and Health Sciences, Notre Dame University, Zouk Mosbeh, P.O.Box: 72, Zouk Mikael, Lebanon; b Afnor Group, Nohra Bldg., Tahwita Highway, Furn El Chebbak. P.O.Box: 16-5806, Beirut, Lebanon; c Human Nutrition Department, College of Health Sciences, QU Health, Qatar University, P.O. Box: 2713, Doha, Qatar

**Keywords:** Food safety culture, Food safety behavior, Food safety management system, Self-assessment tool, COVID-19

## Abstract

The challenges to food safety in Lebanon are many and have worsened due to the Covid-19 pandemic and the Lebanese economic crisis. Against a backdrop of loosely enforced food laws and regulations, a cross-sectional study was carried out in 23 Lebanese food companies on 204 participants using a validated online food safety culture self-assessment tool consisting of 28 indicators. Food safety motivation, burnout/job stress and conscientiousness and their impact on food safety culture were also investigated. Overall, the perceived food safety culture was “good” with a mean value of 119.1 over 140 (equivalent to 4.3/5). A young workforce, the female gender, a science background, and a university degree were associated with a higher food safety culture. The food safety culture score was also perceived higher among participants who attended food safety trainings, and among those working at the managerial level and in the quality department. In addition, the results showed that the food safety culture was significantly better in companies exporting their goods than companies with no international market exposure (121.6 vs 118.1). Moreover, Food safety motivation (mean score 4.1/5) and conscientiousness (3.5/5) were moderately associated with a positive food safety culture. However, the low burnout/job stress scores (2.8/5) may exhibit a negative impact on the food safety culture and could be related to several consequences caused by the Lebanese economic crisis and the Covid-19 pandemic. Further studies are to be conducted to understand better the causal effects relationship.

## 1 Introduction

Foodborne illnesses are a significant cause of morbidity and mortality as well as an impediment to socioeconomic development worldwide. Globally, around 33 million healthy life years (DALYs) are lost due to eating unsafe food; and this number is likely an underestimation [1]. It is becoming apparent that well-elaborated and fit-to-purpose Food Safety Management Systems (FSMS) do not always guarantee food safety nor do national and international food safety-related legislations. This has led to research focusing on human behavior since food poisoning incidents and outbreaks are usually traced back to food handlers’ errors and non-conformance with good working practices [[2], [3], [4]]. Thus, concepts such as food safety climate and culture have emerged [2,[5], [6], [7], [8]].

The notion of safety climate and culture has evolved from the safety and organizational culture studies and the practices in aviation, space, nuclear power, and health care [[9], [10], [11]]. While both food safety climate and food safety culture terminologies are used in the literature [2,12], some authors have used the terms interchangeably [12].

Recently, food safety climate and culture took central stage and became the key ingredient for the needed success of food safety initiatives. Indeed, all the Global Food Safety Initiative recognized certification schemes have added a food safety culture component to be audited and assessed [13]. Furthermore, the Codex Alimentarius Commission, in its 2020 HACCP revision, has introduced the concept of food safety culture [14], and the Food and Drug Administration's New Era of Food Safety advocates a food safety culture in its fourth core element [15]. More recently, the European Union has given food safety culture a permanent place in its regulation (EU) 382/2021 of March 2021 amending the Annexes to regulation (EC) 852/2004 [16]. To ensure simplicity and clarity, this work will adopt the widely recognized terminology of “food safety culture”, as used in all regulatory and standards schemes.

Many assessment and measurement tools and methods have been developed to identify a company's food safety culture based on several relevant dimensions and association with food safety behavior [2,[17], [18], [19], [20], [21], [22], [23], [24]]. These methods can be classified as quantitative, qualitative or mixed-methods approach [2,[25], [26], [27], [28], [29], [30], [31], [32]].).

Recently, Zanin et al. [[33], [34], [35]] worked on educational actions to improve the food safety culture. In addition, Spanioli et al. [36] presented the overall food safety culture diagnosis and proposed the gap analysis methodology, as the first two steps in the food safety culture improvement roadmap.

Moreover, Nyarugwe et al. [37] reported that food safety culture may be influenced by the external political and economic environment. It is worth noting that most of the food safety culture studies were conducted in economically-developed and politically-stable countries, or in transition economies pre-Covid-19 pandemic [20,30,38].

Studies on food safety culture in Lebanon are lacking. The only studies conducted tackle mainly food safety practices in Lebanese food service establishments [39,40] and in hospitals [41,42]. The objective of this work was to assess the perceived food safety culture in Lebanese food companies, during the Lebanese economic crisis and against the backdrop of the Covid-19 pandemic [43]. The food safety culture per company characteristics, participants' job-related parameters, and socio-economic/demographic parameters were also investigated; in addition to the association between the food safety culture and participants’ food safety motivation, burnout/job stress and conscientiousness.

## 2 Material and methods

### 2.1 Study design

A cross-sectional study was carried out - against the backdrop of the Lebanese economic crisis and the Covid-19 pandemic - in order to evaluate the perceived food safety culture in food companies of different types and sizes, across Mount Lebanon, from July 2021 to September 2021, using an online self-assessment questionnaire.

### 2.2 Ethical considerations

The study protocol was approved by the Institutional Review Board of Notre Dame University-Louaize (Protocol Ref#: IRBF2018_1_FNHS). Information on the research objective was introduced to the participants prior filling the survey. The nature of the study was fully explained to respondents and all data were collected after securing consent. Data obtained from participants was treated with confidentiality and the privacy of the respondents was maintained. No false promises such as remuneration, food or financial aids were given. No psychological damage was incurred as the questionnaire was administered online and participants filled it in privacy.

### 2.3 Participants and questionnaire design

Around 48% of Lebanese agri-food companies (total = 1245) are located in Mount Lebanon Governorate [44]. After screening the list of Lebanese food companies obtained from the Ministry of Industry, a convenient sample of 40 food industries were contacted; out of which 23 accepted to enroll. The companies' owners/general managers, or the food safety/quality managers were contacted first via phone calls and then emails. A brief explanation was given about the questionnaire as well as a reassurance of the anonymousness of their answers. Once the approval from the company was received, the questionnaire was send to the available employees including food safety/quality, plant managers, production managers and food handlers/line workers. Participants have signed a consent form prior to filling the online questionnaire (Google Forms) that was sent via email or WhatsApp based on participants’ preferences.

The online questionnaire was composed of two sections. The first section dealt with some socio-economic and demographic parameters that were deemed relevant to the Lebanese circumstances as considered by Nyarugwe et al. [30] and Zabukosek et al. [45]. Also, company characteristics that showed an influence on food safety culture in previous studies were captured using a checklist comprising information about company size, type, presence/absence of a food safety management systems and presence/absence of export activities [18,[20], [21], [22]]. The second section of the questionnaire evaluated the food safety culture using the validated De Boeck et al. [18] self-assessment tool with its 28 indicators. This tool enables the measurement of five components of food safety culture including leadership (six indicators), communication (five indicators), commitment (six indicators), resources (six indicators), and risk awareness (five indicators) [18]. In addition to the mentioned indicators - and in light of the current Lebanese situation and the Covid-19 pandemic and their potential impact on employees’ feelings and behaviors - 5 indicators on food safety motivation, 6 indicators on job stress/burnout and 6 indicators on conscientiousness were also added to the above questionnaire. Some of these additional indicators were based on the study of De Boeck et al. [17]. A five-point Likert answer scale (1 → 5: totally disagree → totally agree) was used for every indicator.

This online questionnaire was made available in both English and Arabic. The questionnaire was translated to Arabic and back-translated to English to test reliability as performed by Martins et al. [46]. Adjustments were made to secure high fidelity during translation and a choice was given to participants regarding language selection. The questionnaire was first pilot tested by 10 adults using face-to-face interviews followed by an online preliminary validation with another group of 30 adults. The clarity, suitability of wording, and the average time needed for its completion (around 20 min) were assessed. Note that the data from the pilot testing were not included in this study.

### 2.4 Statistical analysis

Data were analyzed using IBM® Statistical Package for the Social and Sciences software (SPSS®) version 22 (Chicago, Illinois). The answers’ frequencies and percentages in each category were calculated and tabulated. The correlation between the food safety culture scores and all continuous variables was assessed using Spearman correlation. Further, the relationships between food safety culture and dichotomous or categorical variables were evaluated using independent t-tests or ANOVA/Kruskall-Wallis H tests.

## 3 Results

### 3.1 Characteristics of the recruited food companies

Out of the 40 contacted food companies, 23 agreed to participate in this study, while 17 declined stating reasons like halting operations, downsizing or limited working hours due to Covid-19 measures and/or the economic crisis. Among the 23 companies, 26.1% were micro, 17.4% small, 30.4% medium and 26.1% large as per the Lebanese Ministry of Economy and Trade classification [47]. Around one third (34.8%) were food service establishments (restaurants/diet centers), 30.4% bakeries and confectionary, 17.4% dairy and meat products, and 17.4% canned foods and beverages. Many of the participating companies (65.2%) did not export their products, and 60.1% had a food safety management system implemented (Table 1). Similar studies were conducted elsewhere on varying sample sizes of participating companies ranging from 2 to 136 by De Boeck et al. [17,18], and up to 503 food companies surveyed by Tomasevic et al. [20]. Note that the larger company sample size was only reachable when the study was conducted across borders. Despite the small sample size in the current study; the overall companies’ characteristics were representative in terms of size and type with Lebanese food companies [44]. In fact, the percentage of micro, small and medium enterprises recruited was in line with the official classification published by the Lebanese Chamber of Commerce, Industry and Agriculture; and which represents 79% of the total food industries [48] with food service establishments being the most prevalent, followed by dairy, and then meat and beverage sectors [44]. Note that the olive oil and the wine sectors were not represented in this study, due to the seasonality of their workforce and activities.

**Table 1:** Characteristics of the recruited food companies (n = 23).

	Frequency (n)	Percentage (%)
***Company Size***^a^		
**Micro**	6	26.1
**Small**	4	17.4
**Medium**	7	30.4
**Large**	6	26.1
***Company Type***		
**Food service establishments**	8	34.8
**Bakery/confectionary and cereal**	7	30.4
**Dairy and meat products**	4	17.4
**Canned Food/Beverage**	4	17.4
***Export***		
**Yes**	8	34.8
**No**	15	65.2
***Food Safety Management Systems***^b^		
**Yes**	14	60.1
**No**	9	39.9

a According to the Lebanese Ministry of Economy and Trade: micro enterprise (<LBP 500 million & < 10 employees), small enterprise (<LBP 5 billion & < 50 employees), medium enterprise (<LBP 25 billion & < 100 employees). Exceeding either of these thresholds would lead to recognizing enterprises as large [47].

b ISO 22000, HACCP, FSSC.

### 3.2 Socio-economic and demographic characteristics of participants

The socio-economic and demographic characteristics of employees in the study are presented in Table 2. Among the recruited participants (n = 204) - with a mean age equal to 34.7 ± 8.7 years - 65.2% were males, 91.7% were Lebanese, 51.5% were married, 53.9% had a bachelor's degree, and 28.4% had a degree in sciences/food science/nutrition. Most of them (75.5%) did not have any health conditions. Out of these participants, 45.1% had a monthly net income ranging between 1,500,000–2,999,000 Lebanese Pound (LBP) (ranging from 90 to 187 USD based on the LBP in the black market rate in July 2021; and ranging from 70 to 150 USD in September 2021). The local currency has devaluated between December 2020 till the date of data collection (the LBP has lost more than 90% of its value) and the median take-home income was 1,000,000 LBP (equivalent to 50 USD) [49]. In addition, the study results showed that 85.8% of respondents reported a salary adjustment while 57.8% a salary drop. This ambiguity regarding salaries is attributed to the rapidly devaluating Lebanese currency and hence the meagerness of the value of the salary adjustment/increase provided by the company.

**Table 2:** Socio-economic and demographic characteristics of participants (n = 204).

**Socio-economic & Demographic Characteristics**	**Frequency (n)**	**Percentage (%)**
***Age* (mean ± SD)**	34.7 ± 8.7	
***Gender***		
**Male**	133	65.2
**Female**	71	34.8
***Nationality***		
**Lebanese**	187	91.7
**Other**	17	8.3
***Education Level***		
**Less than high school**	11	5.4
**High school**	58	28.4
**Bachelor**	110	53.9
**Master**	25	12.3
***Major***		
**No major**	73	35.8
**Sciences/FS/Nutrition**	58	28.4
**Other**	73	35.8
***Health Condition***		
**No**	154	75.5
**Yes**	50	24.5
***Monthly Net Income (*LBP)**^a^		
**Less than 749.000**	6	2.9
**750.000–1.499.000**	44	21.6
**1.500.000–2.999.000**	92	45.1
**3.000.000**–**4.499.000**	40	19.6
**More than 4.500.000**	22	10.8
***Salary Adjustment* Yes**	175	85.8
***Salary Drop* Yes**	118	57.8

a The official Lebanese currency had a value fluctuating between 16,000 & 21,000 LBP versus 1 USD between July & September 2021, which was a drop of 11–13 times of its value as compared to pre-crisis.

### 3.3 Participants’ job-related parameters

Job-related parameters were also assessed (Table 3). Results showed that 57.4% of the participants asked for a day off when sick and 48.4% underwent one medical check-up per year. The mean number of years for employees working in the same job was 5.9 ± 4.4. Out of the 204 participants, 10.3% were in managerial positions, 27% worked in the quality/food safety department while 62.7% occupied other positions. 42.2% of the participants had food safety and hygiene trainings more than once per year while 15.2% had none. The participants average working hours per day and per week were 8.4 ± 1.7 h and 46.2 ± 10.6 h, respectively. Furthermore, 55.9% of respondents felt that they might lose their jobs. This feeling of job uncertainty can be explained by the fact that more than half of the Lebanese citizens were below the poverty line during the study period [49]; and the Covid-19 pandemic and its containment measures have pushed 30% of Lebanese into unemployment [[50], [51], [52]].

**Table 3:** Participants’ job-related parameters (n = 204).

Job-related Parameters	**Frequency (n) or mean ± SD**	**Percentage (%)**
***If you are sick?***		
**Ask for a day off**	117	57.4
**Take medication randomly**	13	6.4
**Follow a prescription**	73	35.8
***Frequency of medical checkups per year***		
**One time/month**	3	1.5
**One time/year**	99	48.5
**Two times/year**	36	17.6
**Four times/year**	9	4.4
**Only in case of sickness**	52	25.5
**Never**	5	2.5
***Working hours per day***	8.4 ± 1.7	
***Working hours per week***	46.2 ± 10.6	
***Days off per year***	19.8 ± 10.9	
***Years in current job***	5.9 ± 4.4	
***Felt that you can lose your job***	114	55.9
**Yes**		
***Job role***		
**Managerial position**	21	10.3
**Quality/food safety department**	55	27.0
**Other**^a^	128	62.7
***Frequency of food safety and hygiene training***		
**None**	31	15.2
**More than 1 training/year**	86	42.2
**Yearly**	66	32.4
**Less than 1 training/year**	21	10.3

a Other: Food handlers/line workers.

### 3.4 Food safety culture, food safety motivation, burnout/job stress and conscientiousness scores

The total food safety culture score for all respondents was calculated by adding up all indicator scores (score 1 to 5 for 28 indicators in total, divided into five categories). Table 4 shows that the food safety culture mean score was 119.1 ± 11.1. The range was 84 with a maximum reachable score of 140. The mean scores over 5 ranged between 4.1 and 4.3, with perceptions regarding resources and risk awareness being the lowest. This is translated into a “good” perceived food safety culture. These scores are comparable to that reported by De Boeck et al. [53] among different affiliated butcher shops (n = 23) and farm butcheries (n = 16) with average values of 4.39 and 4.09 respectively. De Boeck et al. [18] also reported that more than 75.3% of the responding companies “agreed” or “totally agreed” that the food safety culture was “good” to “very good” in all 136 Belgian food companies. The food safety culture mean score in this study falls between the ones reported by Tomasevic et al. [20] when comparing food companies in the European Union (EU) (4.36 ± 0.40) to non-EU companies (3.99 ± 0.69). Despite the unstable Lebanese economic situation, the total perceived food safety culture score of 4.3 over 5 points was still satisfactory. This is in contradiction to Nyarugwe et al. [37] who reported that unstable political and economic situations resulted in lower food safety culture scores. It could be explained by the fact that the covid-19 pandemic with its prescriptive preventive measures might have heightened overall hygiene practices - which have resulted in the above food safety culture elevated score [54]. Similarly, the level of knowledge, attitude and practice was satisfactory among Iranian population toward food safety, during covid-19 pandemic [55].

**Table 4:** Food safety culture, food safety motivation, burnout/job stress and conscientiousness scores.

Assessed Items	Mean^a^	SD	Mean/5^b^
1 Leadership	26.2	3.2	4.4
2 Communication	21.7	2.7	4.3
3 Commitment	25.7	3.0	4.3
4 Resources	24.7	2.9	4.1
5 Risk awareness	20.9	2.4	4.2
**Total food safety culture score**	119.1	11.1	4.3
**I believe that workplace hygiene and food safety are important issues to help motivate employees to have better performance.**	4.3	0.5	4.3
**I believe that being involved in all food processing flow of work will help in giving better performance**	4.3	0.6	4.3
**Financial incentives motivate me more than non-financial incentives**	4.1	0.7	4.1
**I am satisfied with the lunch break, rest breaks and leaves given in the organization.**	4.0	0.8	4.0
The salary increments given to employees who do their jobs very well motivates them.	4.1	0.7	4.1
**Food safety motivation**	20.7	2.4	4.1
**I feel mentally exhausted by my job**	2.8	1.2	2.8
**I feel recurrent headaches because of my job**	2.6	1.1	2.6
**I feel I am highly stressed most of the time because of the nature of my job.**	2.7	1.1	2.7
**I feel I cannot enjoy anything anymore.**	2.5	1.1	2.5
**I have to work very fast.**	3.3	1.4	3.3
**I don't have enough time to do everything**	3.2	1.2	3.2
**Burnout/Job stress**	17.0	5.7	2.8
**I pay attention to details**	4.4	0.6	4.4
**I leave where I am working on without cleaning**	1.6	0.9	1.6
**I complete my task completely**	4.4	0.6	4.4
**I do more than what is expected of me**	4.2	0.8	4.2
**I tell the truth if any outbreak happened**	4.5	0.6	4.5
**I often forget to put things back in their proper place**	1.7	1.0	1.7
**Conscientiousness**	20.7	2.3	3.5

a ^The Mean values are obtained from the raw data^.

b ^Mean is the calculated mean value per number of questions with the lowest possible being1 and the highest5^.

Table 4 findings also revealed that the mean score for food safety motivation with its five indicators was 20.7 ± 2.4 (equivalent to 4.1/5), the mean score for burnout/job stress with its six indicators was 17.1 ± 5.7 (equivalent to 2.8/5), and the mean score for conscientiousness with its six indicators was 20.7 ± 2.3 (equivalent to 3.5/5). Although, Tomasevic et al. [20] did not assess the relationship between food safety culture and conscientiousness or feelings of burnout/job stress, it was mentioned as a study limitation. Nascimento et al. [56] on the other hand indicated that mitigating burnout can be an important strategy to improve food safety behavior in the food service industry in Brazil. The low mean score for burnout/job stress in this study could be related to fear of job loss, salary drop, work overload as well as possible work-unrelated stresses (Covid-19 pandemic and the Lebanese economic crisis). To further understand the job stress component, it is suggested to assess work-unrelated stresses and their impact on work behavior. However, it is also essential to acknowledge that the differences in scores among employees may reflect diverse perceptions of the same underlying food safety culture within the organization. For that reason, further associations between participants' socio-economic and demographic characteristics, participants’ job-related parameters, food safety motivation, burnout/job stress, conscientiousness and food safety culture, were assessed.

### 3.5 The association between participants’ socio-economic and demographic characteristics and food safety culture

The association of different socio-economic and demographic variables with food safety culture was investigated using a bivariate analysis (Table 5). Results showed that the perceived food safety culture among males was significantly lower (117.3 ± 9.7) than among females (122.5 ± 12.8, p = 0.003). A negative weak correlation was found between the mean score of food safety culture and age (r = −0.167; p = 0.017), indicating that as age increases the reported food safety culture score decreases. Opposite to the findings of this study, gender did not a have significant effect on food safety culture score as reported by others [17,21,45,57]. There was also a divergence in this study findings regarding age and that of both Olumakaiye and Bakare [57], and Akabanda et al. [58]. These authors showed that older workers had better scores than their younger workmates while Wisniewska et al. [38] reported no significant impact of age on food safety culture in a small franchise restaurant in Poland (18 participants). Regarding education, the study results showed that participants with a high school education had a significantly lower food safety culture score (114.9 ± 6.2) compared to those holding a bachelor's or master's degrees (121.1 ± 10.5, p < 0.001 and 120.6 ± 17.2, p = 0.007, respectively). Furthermore, participants having a degree in science/food science/nutrition had a significantly higher perceived food safety culture mean score (124.1 ± 13.4) than those majoring in other fields (118.8 ± 10.0) or those with no specific major (115.4 ± 8.7). The latter finding is in contrast to that of Zabukosek et al. [45] who established no influence of education on food safety culture in a medium-sized food processing company in Slovenia (220 participants).

**Table 5:** The association between participants’ socio-economic and demographic characteristics and food safety culture.

	Food Safety Culture		
	Mean	SD	p Value
***Gender***			
**Male**	117.3	9.7	**0.003**
**Female**	122.5	12.8	
***Age***	r = −0.16		**0.017**
***Nationality***			
**Lebanese**	118.9	11.1	0.464
**Non-Lebanese**	121.0	12.1	
***Education Level***			
**Less than high school**	117.8	14.9	< **0.001**
**High School**^**a,b**^	114.9	6.2	
**Bachelor**^**a**^	121.1	10.5	
**Master**^**b**^	120.6	17.2	
^**a**^**p < 0.001;**^**b**^**p = 0.007**			
***Major***			
**No major**^**a**^	115.4	8.7	**p < 0.001**
**Sciences/Food science/Nutrition**^**a, b**^	124.1	13.4	
**Other**^**a, b**^	118.8	10.0	
^**a**^**p = 0.013 and p < 0.000;**^**b**^**p = 0.011**			
***Health Conditions***			
**No**	119.4	11.8	0.456
**Yes**	118.2	9.0	
***Monthly net income (LBP)***^a^			
**Less than 749.000**	122.8	13.7	0.126
**750.000–1.499.000**	117.1	9.8	
**1.500.000–2.999.000**	118.2	12.7	
**3.000.000**–**4.499.000**	120.1	7.9	
**More than 4.500.000**	123.9	9.9	
***Salary adjustment***			
**No**	119.6	10.7	
**Yes**	119.0	11.2	0.772
***Salary drop***			
**No**	120.5	13.2	
**Yes**	118.0	9.3	0.140

Significance level set at p < 0.005.

a LBP (Lebanese pound).

### 3.6 The association between participants’ job-related parameters and food safety culture

Table 6 shows that participants working at the managerial level and in the quality/food safety department had significantly higher mean food safety culture scores (125.1 ± 9.50 and 121.6 ± 14.6, respectively) than those having other roles (line workers/food handlers) in the company (117.0 ± 9.0, p < 0.001). De Boeck et al. [19] reported a similar finding where food safety culture differed significantly based on job position and ranking, and Ungku Fatimah et al. [21] described that front-of the-house employees had a more positive perception of the organization's food safety culture than back-of-the-house employees. This implies that a heterogeneous culture exists within an organization, and thus assessment of the food safety culture should take into consideration this important aspect [21,22]. Moreover, in our study, participants who attended more than one food safety and hygiene training per year reported a significantly higher food safety culture mean score compared to those who never attended any training (121.3 ± 13.7 and 118.0 ± 5.9 respectively, p = 0.008). The positive impact of training on food safety culture was already pointed out by several authors [5,8,18,34,37]. This was also the case of a research conducted in 37 hospitals and 24 school canteens, which revealed that employees who had received food safety trainings showed a more positive view regarding food safety culture than untrained employees [21]. Note that, in the guidance document published by the FSSC 22000 Foundation on food safety culture; training is one of the four identified requirements [59]. Yiannas [5] asserted that training is critical albeit in itself will not change behavior; and that even trained employees fail to execute certain tasks based on what they have been taught. Hence, training effectiveness should be backed up by managerial support, positive reinforcement, performance measurement and accountability [60].

**Table 6:** The Association between participants’ job-related parameters and food safety culture.

	Food Safety Culture		
	Mean	SD	P Value
***If you are sick?***			
**Ask for a day off**	120.1	9.0	
**Take medication randomly**	115.8	8.9	
**Follow a prescription**	118.5	13.6	0.305
***Job role***			
**Managerial position**^**a**^	125.1	9.5	
**Quality/food safety department**^**b**^	121.6	14.5	
**Other**^**a,b**^^**a**^**p = 0.001,**^**b**^**p = 0.001**	117.0	9.0	< **0.001**
***Frequency of food safety and hygiene training***			
**None**	118.5	9.8	
**More than 1 training/year**	121.3	13.7	
**Once per year**	119.2	10.9	
**Less than 1 training/year**	118.0	5.9	**0.008**
***Working hours per week***		r = −0.18	**0.008**
***Working hours per day***		r = −0.19	**0.005**
***Days off have per year***		r = 0.17	0.805
***Years in current job***		r = −0.05	0.404
***Felt that you can lose your job***			
**No**	120.9	10.7	
**Yes**	117.6	11.3	**0.035**

Significance level set at p < 0.005.

Other: Food handlers/line workers.

A weak negative correlation was detected between the food safety culture mean score for both the weekly working hours (r = −0.18; p = 0.008) and the daily working hours (r = −0.19; p = 0.005). These results are expected, but no study has previously measured the correlation between working hours and the FSC. Furthermore, the food safety culture mean score was low when participants felt that they could lose their jobs (117.6 ± 11.3) as compared to those who were not worried (120.9 ± 10.7, p = 0.035). Further studies are needed in politically and economically unstable countries in order to draw a better food safety culture picture.

### 3.7 The association between food safety motivation, burnout/job stress, conscientiousness and food safety culture

The food safety motivation and conscientiousness were moderately positively associated with the food safety culture mean score (r = 0.34 and r = 0.28 respectively, p < 0.001) while a negative correlation was found between burnout/job stress (r = −0.37 and p < 0.001) (Table 7). Previously, De Boeck et al. [17] did not identify a dependence between the food safety culture and the level of burnout/job stress, but it was mentioned as a study limitation due to the small size of participating companies (n = 2).

**Table 7:** The association between food safety motivation, burnout/job stress, conscientiousness and food safety culture.

	Food Safety Culture		
	Mean	SD	P Value
**Food safety motivation**	20.7	r = 0.34	**p < 0.001**
**Burnout/job stress**	17.0	r = −0.37	**p < 0.001**
**Conscientiousness**	20.7	r = 0.28	**p < 0.001**

Significance level set at p < 0.005.

Regarding the conscientiousness dimension, a study on 260 employees at a fully-integrated American turkey processing plant reported that both food safety culture and workers’ conscientiousness contributed to the prediction of food safety behaviors; and that individuals having higher traits of conscientiousness presented relatively better food safety behaviors and food safety culture [24]. In fact, conscientiousness is one of the few personality traits that has shown to be a consistent predictor of job performance across different occupations [61]. Conscientiousness is a fundamental personality trait that reflects the tendency to be responsible, organized, hard-working, goal oriented, and to adhere to norms and rules [62].

As for the food safety motivation component, it was shown to have an influence on food safety culture as demonstrated by De Boeck et al. [17] in two Belgian vegetable processing companies. These results show the importance of human factors in the context of food safety culture. In a nutshell, it is obvious that “people” is the critical element for a favorable food safety culture and for decreasing the risk of foodborne illness as stressed in the GFSI position paper. This emphasizes the significance of auditing not just the food safety management system but also the food safety culture, as it represents a crucial and integral component of an effective behavior-based food safety management system. Assessment is the first step to develop and improve food safety culture within the organization [36]. Moreover, to be successful, food safety must go beyond formal regulations to live within the culture of a company [63].

### 3.8 The Association between company characteristics and food safety culture

Regarding companies’ characteristics, further statistical analysis (Table 8) showed that company size (p = 0.109), company type (p = 0.096) and the implementation of food safety management systems (p = 0.050) did not have a significant association with the food safety culture. Likewise, De Boeck et al. [18] did not find a significant correlation between food safety culture and industry type and size. Nevertheless, a better food safety culture was reported in bigger companies [20], among cafeteria employees as compared to those in restaurants [19], in the centrally managed butcher shops and multiple site companies as compared to the independent small scale farm butcheries and single site companies [18,53]. Tomasevic et al. [20] also noted no association of food safety management systems implementation with food safety culture scores when assessing 504 food companies in ten central and eastern European countries. This is in agreement with the results of our study. In fact, Faour-Klingbiel et al. [40] reported that companies with proper resources and well-established food safety management systems provide better food safety environments but do not necessarily have a good food safety culture or risk awareness. Furthermore, the current study showed a higher mean score (121.6 ± 10.7) for exporting companies compared to companies that did not export (118.1 ± 11.2; p = 0.043). This could be attributed to the fact that these companies have a better financial performance through the flow of hard currency despite the Lebanese economic crisis. Therefore, better resources, better job security with a lower likelihood of job stress or fear of job loss.

**Table 8:** The association between company characteristics and food safety culture.

	Food Safety Culture		
	**Mean**	**SD**	**P value**
***Company size***			
**Micro**	118.5	7.1	
**Small**	123.8	11.2	
**Medium**	119.4	13.0	
**Large**	117.7	10.6	0.109
***Company type***			
**Food service establishments**	120.1	11.5	
**Fish/dairy/poultry**	119.4	10.9	
**Bakery/confectionary**	116.6	10.8	
**Canned food/beverage**	122.2	10.5	0.096
***Export***			
**No**	118.1	11.2	
**Yes**	121.6	10.7	**0.043**
***Food safety management systems***			
**None**	120.4	8.8	
**Yes**	119.1	11.7	0.050

## 4 Limitations

This study provides valuable information on the current food safety culture in Lebanese food companies amid an unprecedented economic crisis and the Covid-19 pandemic. Several limitations are recognized in the study design. First, the survey was self-administered, which would require the food worker to be able to read. The second limitation is that companies choosing to participate can be considered to be already food safety-oriented, so there might be some partiality in the sample selection. Third, the use of a self-reported assessment of food safety culture could have produced a biased result as respondents may have provided socially-desirable answers despite the guarantee of confidentiality [64]. In addition, the validity of this developed tool needs to be assessed for the Arab culture [31] and single-method (self-reported survey ratings) derived results could lead to wrong conclusions [19,28]. These limitations should be taken into account, and interpretations of the findings done with caution. Overall, understanding the complexities surrounding food safety culture assessments and comparisons is critical to ensure that research in this area contributes to improving food safety practices effectively.

## 5 Conclusions

This investigation provided a valuable insight on the food safety culture in Lebanese food companies amid the Covid-19 pandemic and the Lebanese economic crisis. Overall, the total mean score of food safety culture was perceived to be “good”. Based on our findings, Lebanese food companies need to consider the following factors when working on improving the perceived food safety culture, such as employees’ educational background and trainings, proper working hours, minimizing job stress/burnout, and providing job security/stability.

A cohort study is suggested to evaluate Lebanese food companies emerging from the economic crisis and the Covid-19 pandemic. To the best of our knowledge, no studies are available in the Arab region on food safety culture in food companies. In addition, no recent studies have been performed to assess the impact of Covid-19 pandemic on food safety culture in food companies across the globe. Further research is also needed to adopt a comprehensive mixed-methods approach to assess food safety culture and its impact on food safety outcome. It would be also interesting to study the multidimensional connotations of the concept of food safety culture within these organizations. This would assess whether the food safety culture concept can be further subdivided into distinct elements, such as food safety system culture, food safety behavior culture, food safety concept culture, and food safety physical culture.

In conclusion, food safety culture research and recommendations are mainly based on working groups and studies performed in developed Western countries. The introduced elements and dimensions of food safety culture might not capture the rest of the world and hence be biased as values, beliefs and norms vary between cultures and regions. Further research could also investigate food safety culture in countries operating with different food safety governance approaches and national cultures.

## Funding

This research did not receive any specific grant from funding agencies in the public, commercial, or not-for-profit sectors. Open access funding was provided by the Qatar National Library.

## Ethics statement

The study complies with all regulations and informed consent was obtained from the participants in collecting the samples.

## Author contribution statement

Zeina Nakat, Christelle Bou-Mitri, Layal Karam: Conceived and designed the experiments; Analyzed and interpreted the data; Wrote the paper.

Vera Tayoun: Conceived and designed the experiments; Performed the experiments; Analyzed and interpreted the data; Wrote the paper.

Samar Merhi: Conceived and designed the experiments; Analyzed and interpreted the data; Contributed reagents, materials, analysis tools or data; Wrote the paper.

## Data availability statement

Data will be made available on request.

## Declaration of competing interest

The authors declare that they have no known competing financial interests or personal relationships that could have appeared to influence the work reported in this paper.
